# Integrated Targeted and Untargeted Metabolomics Reveals the Toxic Mechanisms of Zearalenone in Goat Leydig Cells

**DOI:** 10.3390/ani16020283

**Published:** 2026-01-16

**Authors:** Chunmei Ning, Jinkui Sun, Ying Zhao, Houqiang Xu, Wenxuan Wu, Yi Yang

**Affiliations:** 1 Key Laboratory of Animal Genetics, Breeding and Reproduction in the Plateau Mountainous Region, Ministry of Education, Guizhou University, Guiyang 550025, China; 18303619050@163.com (C.N.); s1907205810@163.com (J.S.); 18085681238@163.com (Y.Z.); gzdxxhq@163.com (H.X.); wxwu@gzu.edu.cn (W.W.); 2 College of Animal Science, Guizhou University, Guiyang 550025, China; 3 Institute of New Rural Development, Guizhou University, Guiyang 550025, China

**Keywords:** goat, Leydig cells, cell apoptosis, targeted metabolomics, untargeted metabolomics

## Abstract

Zearalenone (ZEA) is a mycotoxin commonly found in animal feed and is associated with pronounced reproductive toxicity. However, most studies on ZEA’s reproductive effects have focused on female monogastric animals, while research on male ruminants remains limited. This study aimed to investigate the cytotoxic and metabolic mechanisms underlying ZEA-induced damage in goat Leydig cells (LCs). The CCK8 assay was first used to determine the effective ZEA concentration (IC_50_ ≈ 20 μM), and a cytotoxicity model was subsequently established. The model’s validity was confirmed using qRT-PCR, transmission electron microscopy, flow cytometry, and JC-1 staining. Results showed that ZEA significantly reduced LCs viability in a dose-dependent manner, decreased mitochondrial membrane potential, induced cell cycle arrest, and triggered apoptosis. Targeted and untargeted metabolomics analyses revealed that ZEA disrupts steroidogenic pathways and alters steroid hormone secretion, resulting in elevated levels of progesterone, corticosterone, and androstenedione, and reduced dihydrotestosterone levels. Furthermore, 52 significantly altered metabolites were identified, predominantly enriched in glycerophospholipid metabolism, choline metabolism, and neurotransmitter vesicle pathways, with corresponding changes in gene expression. Collectively, this study has confirmed that ZEA causes harm to the reproductive cells of male goats in multiple aspects, underscoring the link between metabolic dysregulation and reproductive impairment, and offering a foundation for evaluating ZEA’s impact on goat reproductive performance.

## Simple Summary

This study focuses on the toxic mechanisms of zearalenone (ZEA) in goat Leydig cells (LCs). Using various experimental techniques, it is found that ZEA damages LCs in a dose-dependent manner, induces cell apoptosis and cycle arrest, and disrupts steroid hormone synthesis, as well as metabolic pathways such as glycerophospholipid and choline metabolism. This study clarifies the multifaceted hazards of ZEA to male goat reproductive cells and provides theoretical support for evaluating the impact of ZEA on goat reproductive performance.

## 1. Introduction

The goat industry represents a vital sector of animal husbandry, with reproductive performance serving as a key determinant of the efficiency and productivity of the goat industry. Regulation of male reproductive function plays a pivotal role in enhancing reproductive efficiency, particularly through the maintenance of testosterone homeostasis. Testosterone, primarily synthesized by testicular Leydig cells (LCs), is essential for sex differentiation, spermatogenesis, and the maintenance of male secondary sexual characteristics [1]. Abnormal testosterone levels can result in male reproductive disorders, such as reduced semen quality and increased sperm malformation rates, and are also strongly associated with diseases including adrenal tumors and cortical hyperplasia [2,3,4]. Therefore, maintaining stable testosterone levels is critical for preserving male reproductive function and overall health.

In recent years, mycotoxin contamination in animal feed has become a critical factor limiting the health and productivity of livestock and poultry. Among various mycotoxins, ZEA is of particular concern due to its chemical stability and resistance to degradation. ZEA is widely present in corn, sorghum, and animal feed, and is regarded as one of the most hazardous fungal toxins in animal husbandry, posing a serious threat to human safety and animal health [5,6]. Numerous studies have shown that ZEA and its metabolites can induce reproductive disorders, disrupt immune and endocrine system functions, cause visceral organ damage, and even promote tumor development [7,8,9]. Although ZEA can act on multiple tissues and organs, the reproductive system is recognized as its primary target [10,11]. Because its molecular structure is closely resembles that of estrogen, ZEA competitively binds to estrogen receptors (ER) and forms ZEA–ER complexes, which is translocated into the nucleus. This interaction disrupts the transcriptional regulation of reproduction-related genes, leading to reproductive dysfunction, organ abnormalities, and impaired fertility in animals [12,13,14]. In female animals, ZEA exposure often results in impaired corpus luteum formation, ovarian atrophy, and uterine abnormalities, and in severe cases, can cause abortion, premature birth, or stillbirth [15,16,17]. At the cellular level, ZEA significantly reduces the viability of endometrial stromal cells (ESCs) and ovarian granulosa cells (GCs), inducing apoptosis, necrosis, and oxidative stress responses [18,19,20]. Moreover, ZEA exposure further disrupts GCs by causing cell cycle arrest and intensifying oxidative stress [21].

In addition, owing to its estrogen-like effects, ZEA has the physiological potential to disrupt steroid hormone synthesis and exert toxic effects on the male reproductive system. Previous studies have demonstrated that ZEA induces structural abnormalities in testicular tissue, reduces sperm motility, and lowers testosterone levels [22,23]. Boeira et al. reported that oral administration of ZEA (40 mg/kg for 48 h) in mice significantly decreased sperm count, motility, and testosterone concentrations [24]. Similarly, Yan et al. [25] showed that 57.5 μM ZEA induced apoptosis and oxidative stress in porcine testicular cells, thereby reducing their antioxidant capacity. ZEA has also been shown to promote reactive oxygen species (ROS)-mediated oxidative stress and autophagy in goat Sertoli cells [26]. Furthermore, the toxic effects of ZEA on male animals can be transmitted to offspring through impaired sperm quality, resulting in increased sperm malformation rates and reduced fertility [27].

However, most studies on the toxic effects of ZEA have focused on monogastric animals or females, whereas research on male ruminants, particularly testicular LCs, remains limited, and the underlying mechanisms are not yet fully understood. Considering that LCs are the principal cell type responsible for steroid hormone synthesis, this study employed goat Leydig cells as a model and utilized both targeted and untargeted metabolomic approaches to systematically investigate the toxic effects of ZEA and its impact on metabolic pathways. The objective was to elucidate the mechanisms underlying ZEA-induced toxicity and to provide a theoretical basis for developing effective strategies to mitigate and degrade ZEA.

## 2. Materials and Methods

### 2.1. Experimental Animals and Sampling

Testes were collected from three healthy, one-month-old Guizhou Black goats raised at the Experimental Goat Farm of the College of Animal Science, Guizhou University. All animal housing, management, and sample collection procedures were approved by the Animal Ethics Committee of Guizhou University (Approval No. EAE-GZU-2020-T064) and conducted in strict accordance with institutional animal welfare and ethical guidelines.

### 2.2. Isolation, Culture and Identification of Testicular LCs

These animals were anesthetized with atropine sulfate salt monohydrate (0.04 mg/kg) (A0257; Sigma-Aldrich, St. Louis, MO, USA) before sampling. Anesthesia and surgical procedures were performed by a veterinarian, then the testicles were collected. The testes samples were digested in Dulbecco’s modified Eagle’s medium/F-12 (DMEM/F-12) (Gibco, Waltham, MA, USA) containing 10% fetal bovine serum (FBS) (Thermo Fisher Scientific, Waltham, MA, USA) and 1 mg/mL type I collagenase (C8140, Beijing Solarbio Science & Technology, Beijing, China) at 37 °C for 1 h by constant stirring. The collagenase digestion was terminated with a medium containing 10% FBS, filtered through 200 and 400 mesh cell sieves and centrifuged at 800× *g* for 10 min. The LCs were collected from precipitation and cultured in DMEM/F-12 containing 10% FBS at 37 °C with 5% CO_2_. Cell purity was evaluated by indirect immunofluorescence using 3β-HSD (Proteintech, 10366-1-AP, Suzhou, China) as the primary antibody and Alexa Fluor 488 (Proteintech, SA00006-2, Suzhou, China) as the secondary antibody. The cells were fixed with 4% paraformaldehyde, washed with PBS, and then permeated with 0.5% Triton X-100 for 10 min. After PBS washing, they were blocked with 5% BSA for 30 min. After PBS washing, the first antibody was incubated at 4 °C overnight, and then the second antibody was incubated in the dark at 37 °C. After PBS washing, the DAPI staining solution (10 μg/mL) was added for 10 min, and finally the cells were observed under a microscope (Nikon Eclpse Ti5, Tokyo, Japan).

### 2.3. Cell Counting Kit-8 (CCK-8) Analysis

Cells were digested with 0.25% trypsin and resuspended to a density of 3 × 10^4^ cells/mL. A volume of 100 μL of the cell suspension was seeded into each well of a 96-well plate and incubated for 12 h. Experimental groups included a blank group (basal medium), a control group (basal medium + cells), and ZEA treatment groups (5, 10, 20, and 30 μM), with three replicates per group. After 24 h of ZEA exposure, 10 μL of CCK-8 solution was added to each well, and the plate was incubated for an additional 2 h. Absorbance at 450 nm was then measured, and cell viability was calculated. The half-maximal inhibitory concentration (IC_50_) of ZEA was also determined.

(1) $$Cell\;survival\;rate\left(\%\right)=\left[\frac{Experimental\;group\;OD-Blank\;group\;OD}{Control\;group\;OD-Blank\;group\;OD}\right]\times 100\%$$

### 2.4. Electron Microscopy Observation

After treatment with ZEA (0 μM, 20 μM) for 24 h, LCs were harvested following digestion with 0.25% trypsin. The cells were centrifuged to remove the supernatant (3 min, 800× *g*), and 1 mL of 2.5% glutaraldehyde was gently added for fixation at 4 °C for 24 h. Cells were then washed with pre-cooled PBS and dehydrated using graded ethanol solutions, followed by acetone treatment. Subsequently, cells were post-fixed in 1% osmium tetroxide for approximately 2 h. Embed the cell clusters with epoxy resin. During embedding, ensure that the key areas of the sample and the direction of the section are perpendicular. Then, the sections are successively stained with uranyl acetate (for 5 min) and lead citrate (for 15 min). Finally, the ultrastructure of the cells was examined using a transmission electron microscope (TEM) (ZEISS, GeminiSEM 450, Oberkochen, Germany).

### 2.5. Real-Time Quantitative PCR

After treatment with ZEA (0 μM, 20 μM) for 24 h, LCs were collected, the supernatant discarded, and washed with PBS. Total RNA was extracted from the cells using an RNA extraction kit (Yisen, Shanghai, China). Add 1 mL of lysis buffer LB to lyse the cells, and let it stand at room temperature for 5 min. Then add 0.2 mL of chloroform and perform centrifugation to obtain the supernatant (12,000× *g*, 4 °C, 10 min). Add 0.5 mL of anhydrous ethanol to the supernatant and mix well. The mixture is filtered through column A1 of RNA adsorption, and then 0.5 mL of protein removal solution PL and 0.5 mL of washing solution are added successively. Finally, 50 μL of RNase-free H_2_O is added. Centrifuge at 12,000× *g* for 1 min to obtain the RNA solution. RNA concentration was measured using a NanoDrop 2000 spectrophotometer (Thermo Fisher Scientific, Waltham, MA, USA). First-strand cDNA was synthesized using the RevertAid First-Strand cDNA Synthesis Kit (Thermo Fisher Scientific, Waltham, MA, USA). qRT-PCR was performed using PowerUp SYBR Green Master Mix (Thermo Fisher Scientific, Waltham, MA, USA) in a 10 µL reaction volume on a C1000 Touch Thermal Cycler (Thermo Fisher Scientific, Waltham, MA, USA). Each group included three biological replicates. Cycling conditions were: initial denaturation at 95 °C for 5 min, followed by 34 cycles of 95 °C for 30 s, annealing at 60 °C for 60 s, and extension at 72 °C for 60 s. β-actin was used as the reference gene, and relative gene expression was calculated using the 2^−ΔΔCt^ method. Primer sequences for the target and reference genes are listed in Table 1.

### 2.6. Cells Healing Ability Test

LCs were treated with ZEA (0 μM, 20 μM) for 24 h. When cell confluence reached approximately 90%, a straight scratch was made in each well using a 10 µL pipette tip. Cells were then washed with PBS to remove debris, and 10% complete culture medium was added. Cell migration and wound closure were observed and photographed under a microscope 18 h later.

(2) $$Cell\;migration\;rate\left(\%\right)=\left[\frac{Initial\;area-Key\;area}{Initial\;area}\right]\times 100\%$$

### 2.7. Assessment of Mitochondrial Membrane Potential Using JC-1 Assay

After treatment with ZEA (0 μM, 20 μM) for 24 h, LCs were washed twice with PBS and 1 mL of culture medium was added. Subsequently, 1 mL of JC-1 working solution was added, and the cells were gently mixed and incubated in the dark in a cell culture incubator for 20 min. The plate was gently shaken every 10 min to ensure even distribution of the probe. After incubation, the supernatant was removed, and the cells were washed twice with 1 × JC-1 staining buffer. Fluorescence images were then captured using a fluorescence microscope (Nikon Eclpse Ti5, Tokyo, Japan).

(3) $$Membrane\;potential\;level=\left[\frac{Red\;fluorescence\;intensity}{Green\;fluorescence\;intensity}\right]$$

### 2.8. Flow Cytometric Cycle Assay

After treatment with ZEA (0 μM, 20 μM) for 24 h, LCs were digested with 0.25% trypsin and collected. The cells were centrifuged to remove the supernatant (3 min, 800× *g*), washed with PBS, and centrifuged again. The cell pellet was then fixed overnight at 4 °C in 2 mL of 70% ethanol. Centrifugation at 800× *g* for 5 min to remove the supernatant, cells were washed with PBS and the supernatant discarded, 500 μL of PI staining solution was added, gently blown and the cells were mixed, incubated at 37 °C in the dark for 30 min (Servicebio, Wuhan, China). Cell cycle distribution was measured using a flow cytometer (CytoFLEX, Beckman, Brea, CA, USA).

### 2.9. Targeted Metabolomics Analysis

LCs were treated with ZEA (0 μM, 20 μM) for 24 h and collected. For each 200 μL LC sample, 20 μL extraction solvent A, 200 μL methanol, and 200 μL ultrapure water were added, thoroughly mixed, and centrifuged. A 450 μL aliquot of the supernatant was applied to an activated SPE plate. Sequential washes were performed with 200 μL extraction solvent B and 200 μL n-hexane, followed by elution of the target compounds using 60 μL extraction solvent C. All eluates were collected in a 96-well plate, and 75 μL ultrapure water was added to each well. Samples were mixed and loaded into an autosampler for analysis. HPLC Conditions: Samples were separated using an ultra-high-performance liquid chromatography (UHPLC) system. The mobile phase consisted of solvent A (ammonium fluoride in water) and solvent B (methanol). Samples were maintained at 4 °C in the autosampler, with the column temperature set to 40 °C, a flow rate of 100 μL/s, and an injection volume of 25 μL. The mobile phase gradient was 45–95–45% B over 15 min. Low-, medium-, and high-concentration quality control (QC) samples were included to ensure system stability and reproducibility. MS Parameters: Mass spectrometric analysis was conducted in both positive and negative ion modes using an ESI source. Source temperature was set to 600 °C; Gas1 and Gas2 at 60 psi; curtain gas (CUR) at 35 psi; and ion spray voltage (ISVF) at −4500 V for negative ions and 5500 V for positive ions. Target ions were detected using multiple reaction monitoring (MRM) mode.

### 2.10. Untargeted Metabolomics Analysis

Cell samples were mixed with pre-cooled methanol/acetonitrile/water (2:2:1, *v*/*v*/*v*), vortexed, and subjected to low-temperature sonication for 30 min. After incubation at −20 °C for 10 min, the samples were centrifuged, and the supernatant was vacuum-dried. The dried residue was reconstituted in 100 μL acetonitrile/water (1:1, *v*/*v*), vortexed thoroughly, centrifuged, and the resulting supernatant was subjected to mass spectrometry analysis. UPLC Conditions: Separation was performed on a column maintained at 25 °C, with a flow rate of 500 μL/min and an injection volume of 2 μL. The mobile phase consisted of solvent A (water containing 25 mM ammonium acetate and 25 mM ammonia) and solvent B (acetonitrile). The gradient program was as follows: 0–30 s, 95% B; 30 s–7 min, 95–65% B; 7–8 min, 65–40% B; 8–9 min, 40% B; 9–9.1 min, 40–95% B; 9.1–12 min, 95% B. Samples were stored at 4 °C and injected in a randomized order to minimize signal variability. MS Conditions: Both MS^1^ and MS^2^ spectra were acquired in positive and negative electrospray ionization (ESI) modes. ESI source parameters were: source temperature 600 °C, Gas1 and Gas2 at 60 psi, curtain gas (CUR) 30 psi, and ion spray voltage (ISVF) ±5500 V. MS acquisition parameters were: MS^1^ scan range *m*/*z* 60–1000 (0.20 s per spectrum) and MS^2^ scan range *m*/*z* 25–1000 (0.05 s per spectrum). MS^2^ spectra were obtained using information-dependent acquisition (IDA) in high-sensitivity mode, with a declustering potential (DP) of ±60 V and collision energy of 35 ± 15 eV. IDA settings included exclusion of 4 Da isotopes and monitoring of 10 ions per cycle.

### 2.11. Statistical Analysis

Between-group comparisons were performed using SPSS 21.0 software (SPSS, Inc., Chicago, IL, USA), with one-way ANOVA followed by the least significant difference (LSD) post hoc test or independent samples *t*-test and further validated the results using Tukey’s honestly significant difference (HSD) test. Data are shown as mean ± SD from three independent experiments, each performed in technical triplicate (n = 3). Differences between the two groups are indicated by * for *p* < 0.05 (relative to the untreated cells) and ** for *p* < 0.01 (relative to the untreated cells).

## 3. Results

### 3.1. Cell Purity Identification

**Figure 1:**
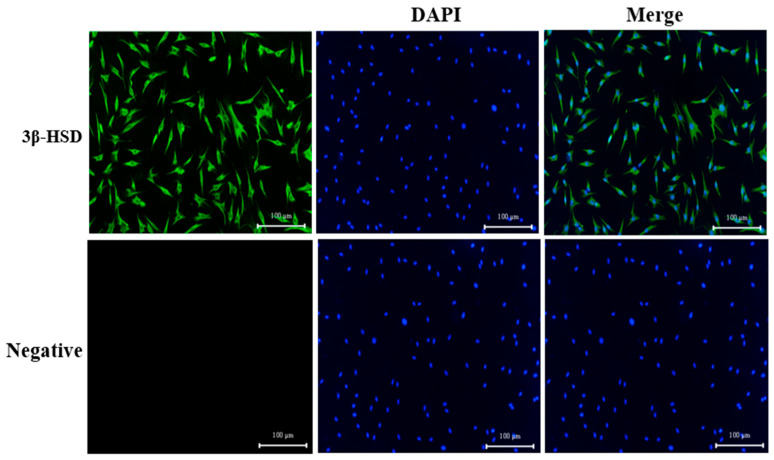
Identification of LCs. Indirect immunofluorescence was used to detect the degree of binding of LCs to anti-Alexa Fluor488 (Green, 1:500, Suzhou, China); nuclei were stained with DAPI (Blue). Note: Bar = 100 μm. n = 3.

The purity of LCs was evaluated using an indirect immunofluorescence method. The results indicated that LCs emitted green fluorescence when bound to the secondary antibody (Goat anti-Rabbit IgG (H + L), Alexa Fluor 488), while unbound cells exhibited no fluorescence (Figure 1). The complete overlap of cytoplasmic and nuclear staining confirmed the identity of the cells as LCs.

### 3.2. Effects of ZEA on LCs Viability

**Figure 2:**
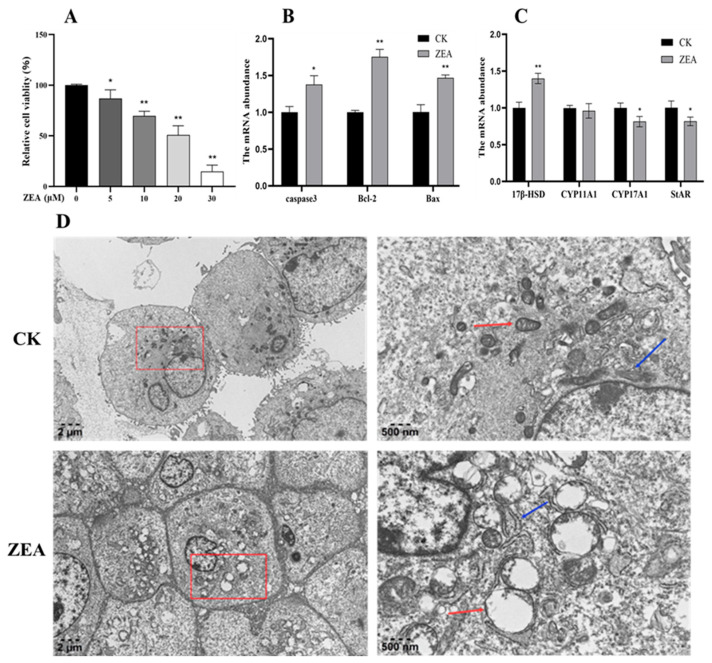
The effect of ZEA on goat LCs. (**A**) Viability changes in LCs after treated with increasing concentrations of ZEA. (**B**) The effect of ZEA on the LCs apoptosis genes; (**C**) The influence of ZEA on the LCs testosterone synthesis genes; (**D**) The influence of ZEA on the ultrastructure of LCs (The picture on the right is the enlarged red box in the left picture. The blue arrows indicate the endoplasmic reticulum, and the red arrows indicate the mitochondria). Note: Bars represent mean ± SD (n = 3). CK: Control check; ZEA: Zearalenone. The same below. * for *p* < 0.05 (relative to the CK) and ** for *p* < 0.01 (relative to the CK).

Treatment of LCs with increasing concentrations of zearalenone (ZEA) resulted in a dose-dependent reduction in cell viability (Figure 2A). Compared with the control group (0 μM ZEA), all ZEA-treated groups showed a significant decrease in viability. Specifically, 10 μM ZEA caused a highly significant reduction (*p* < 0.01), 20 μM ZEA decreased viability by approximately 50%, and 30 μM ZEA led to near-complete cell death. The half-maximal inhibitory concentration (IC_50_) of ZEA was estimated to be 20 μM, which was subsequently used for further experiments.

### 3.3. Effects of ZEA on mRNA Expression of Genes Related to LCs

qRT-PCR was conducted to assess the mRNA expression levels of apoptosis-related genes (*Bcl-2*, *Bax*, and *Caspase3*) and key genes involved in testosterone biosynthesis (*StAR*, *CYP17A1*, *CYP11A1*, and *17β-HSD*) in control and ZEA-treated groups. Compared with the control group, ZEA treatment significantly increased the mRNA expression of *Bcl-2*, *Bax*, and *Caspase3* (*p* < 0.05) (Figure 2B). In contrast, the mRNA levels of *StAR* and *CYP17A1* were significantly decreased (*p* < 0.05), whereas *17β-HSD* mRNA expression was significantly increased (*p* < 0.05). No significant change was observed in *CYP11A1* mRNA expression (Figure 2C). These results indicate that ZEA treatment promotes the expression of apoptosis-related genes in LCs and disrupts the expression of key components in the testosterone biosynthesis pathway, ultimately impairing testosterone production.

### 3.4. ZEA Affects the Ultrastructure of the LCs in Goats

In the control group, cells displayed a plump morphology with intact plasma membranes and well-defined nuclear envelopes. Mitochondrial cristae were neatly arranged, with no evidence of disorganization, swelling, or rupture. The mitochondrial structure remained stable, the endoplasmic reticulum (ER) was continuous without fragmentation, and chromatin was uniformly distributed. Owing to rapid cell proliferation and adherence, a few autophagosomes were occasionally observed. In contrast, ZEA-treated LCs exhibited pronounced organelle damage, including ER fragmentation, indistinct structural features, severe mitochondrial vacuolization, formation of autophagic vesicles, and chromatin condensation (Figure 2D).

### 3.5. ZEA Impairs the Wound-Healing Ability of LCs

**Figure 3:**
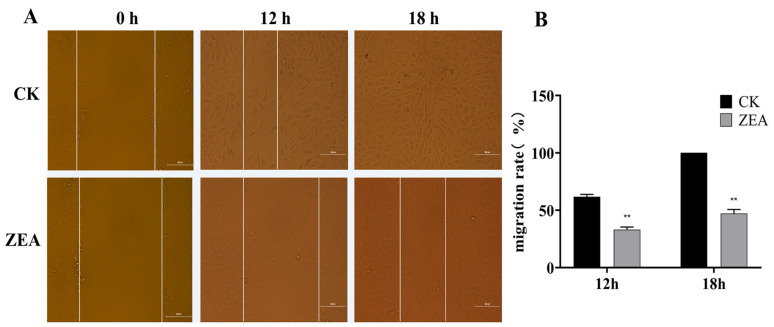
Effect of ZEA treatment on the healing ability of LCs. (**A**) Representative photomicrographs of the cells and impact on gap width; (**B**) Cell migration rate. Note: Bar = 100 μm. ** for *p* < 0.01 (relative to the CK).

The scratch assay results showed that cell migration was significantly inhibited (Figure 3). At 0 h, the scratch widths were similar between the control and ZEA-treated groups. After 12 h of incubation, control cells had nearly fused, reaching a wound-healing rate of approximately 50%, whereas ZEA-treated cells exhibited only about 20% healing. By 18 h, the control group achieved complete wound closure, while the ZEA group reached a healing rate of about 80% but did not fully close. These findings indicate that ZEA treatment markedly reduces the wound-healing capacity of LCs.

### 3.6. Effects of ZEA on Mitochondrial Membrane Potential in LCs

**Figure 4:**
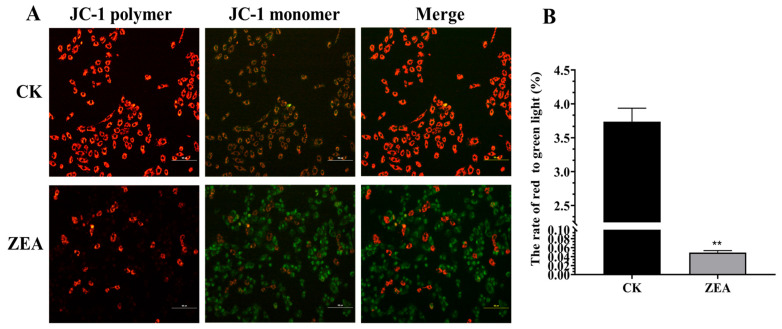
Effect of ZEA treatment on the mitochondrial membrane potential level of LCs. (**A**) The luminescence of mitochondrial membrane potential; (**B**) Membrane potential level (The mitochondrial membrane potential level is represented by the ratio of red to green fluorescence). Note: Bar = 100 μm. Bars represent mean ± SD (n = 3). ** for *p* < 0.01 (relative to the CK).

JC-1 staining revealed that mitochondria in the control group predominantly exhibited red fluorescence, indicating high polarization and membrane potential. Following ZEA treatment, red fluorescence decreased while green fluorescence increased markedly, indicating mitochondrial depolarization and a reduction in membrane potential (Figure 4).

### 3.7. ZEA Affects the Cell Cycle of Goat LCs

**Figure 5:**
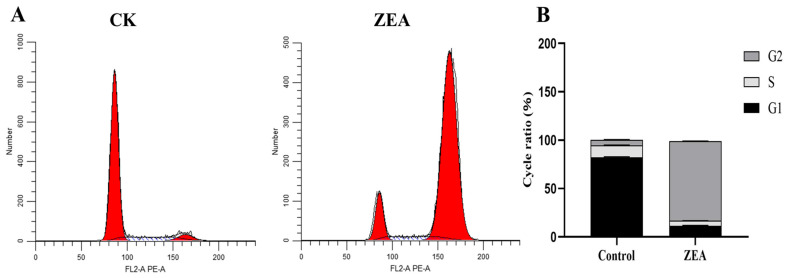
Effect of ZEA on cell cycle progression of LCs. (**A**) Cell Cycle; (**B**) Cell cycle ratio. G1: Early stage of DNA synthesis; S: DNA synthesis phase; G2: Late stage of DNA synthesis. Note: Bars represent mean ± SD (n = 3).

Flow cytometric analysis of cell cycle progression in LCs following ZEA treatment revealed (Figure 5). In the control group, 82.48% of cells were in G1 phase, 12.15% in S phase and 5.37% in G2 phase. After ZEA exposure, the proportions of cells in G1 and S phases decreased significantly (*p* < 0.05) to 11.13% and 5.58%, respectively, while the G2 phase population increased markedly (*p* < 0.05) to 82.39%. These results indicate that ZEA disrupts cell cycle progression in LCs, inducing cell cycle arrest at the G2 phase.

### 3.8. Targeted Metabolomics Study of ZEA Effects on Goat LCs

**Figure 6:**
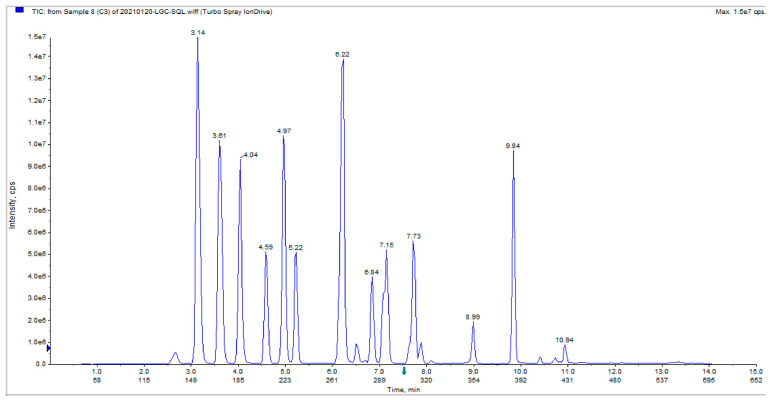
Total ion chromatograms (TIC) diagram of the steroid standard mixture.

The total ion chromatograms (TICs) of steroid metabolite standards showed well-resolved peaks with sharp and symmetrical shapes, indicating that the method is suitable for the quantitative analysis of various steroid hormones (Figure 6). ZEA treatment altered the secretion levels of steroid hormones in goat LCs. Compared with the control group, treatment with ZEA (20 μM, 24 h) significantly increased the levels of 17α-hydroxyprogesterone, corticosterone, progesterone, 21-hydroxyprogesterone, androstenedione, and cortisone (*p* < 0.05). Aldosterone levels also increased, although the difference was not statistically significant. In contrast, dihydrotestosterone levels were significantly reduced (*p* < 0.05), while estradiol levels showed a slight but non-significant decrease (Table 2). These results indicate that ZEA disrupts steroid hormone metabolism in goat LCs.

### 3.9. Untargeted Metabolomic Investigation of ZEA Effects on Goat LCs

**Figure 7:**
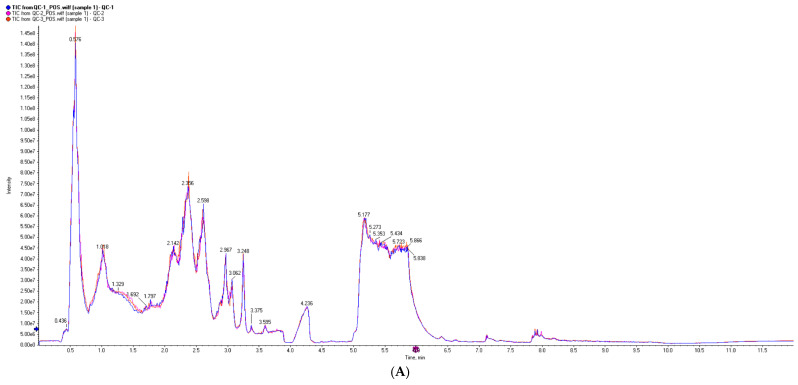

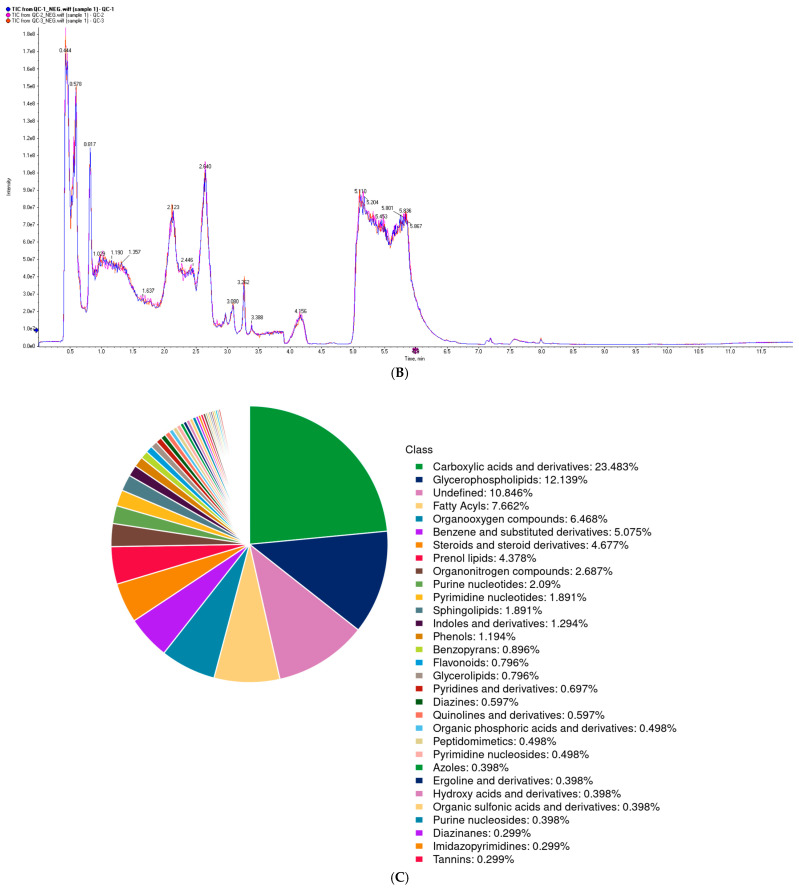
Total ion chromatogram (TIC) and metabolite classification. (**A**) Positive ion chromatogram; (**B**) Negative ion chromatogram; (**C**) Ratio of metabolite quantities. Note: The horizontal axis represents the retention time of each chromatographic peak, while the vertical axis indicates the intensity value of the peak.

**Figure 8:**
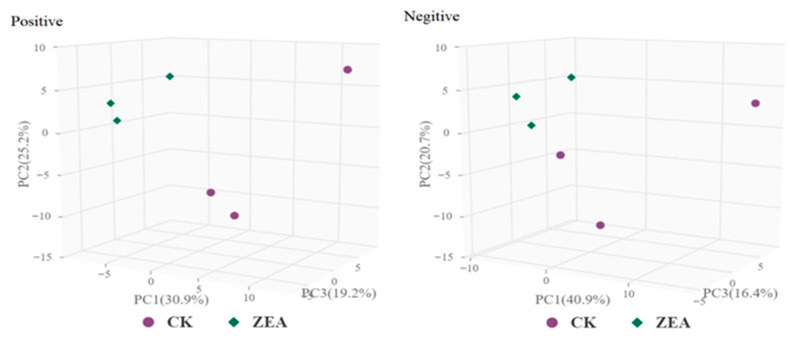
Principal component analysis (PCA) score map of samples.

**Figure 9:**
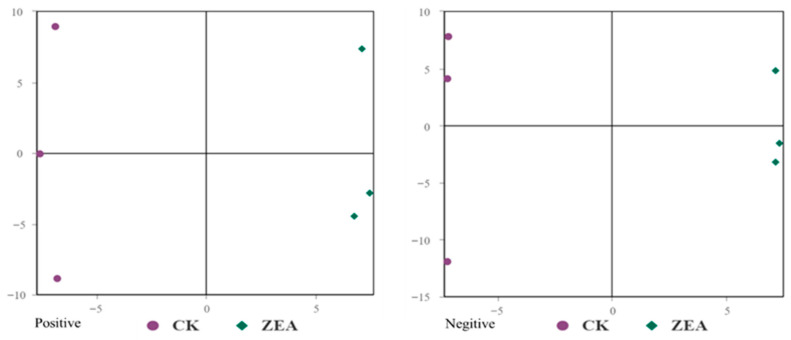
Orthogonal partial least squares discriminant analysis (OPLS-DA) score map.

**Figure 10:**
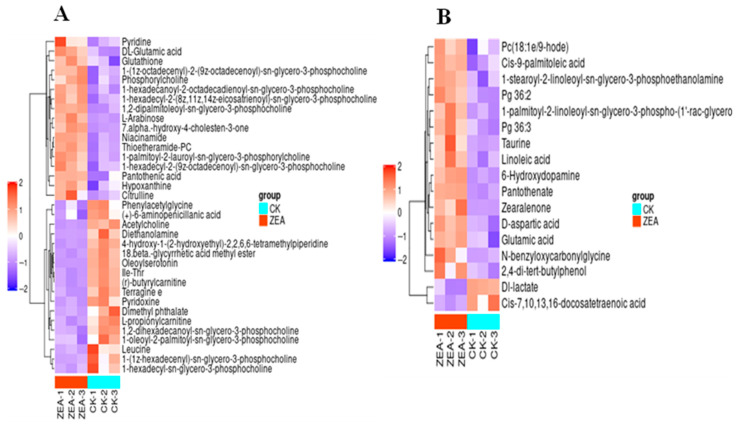
Heat map clustering of significant metabolite on positive and negative ion modes. (**A**) Positive ion heatmap; (**B**) Negative ion heatmap.

**Figure 11:**
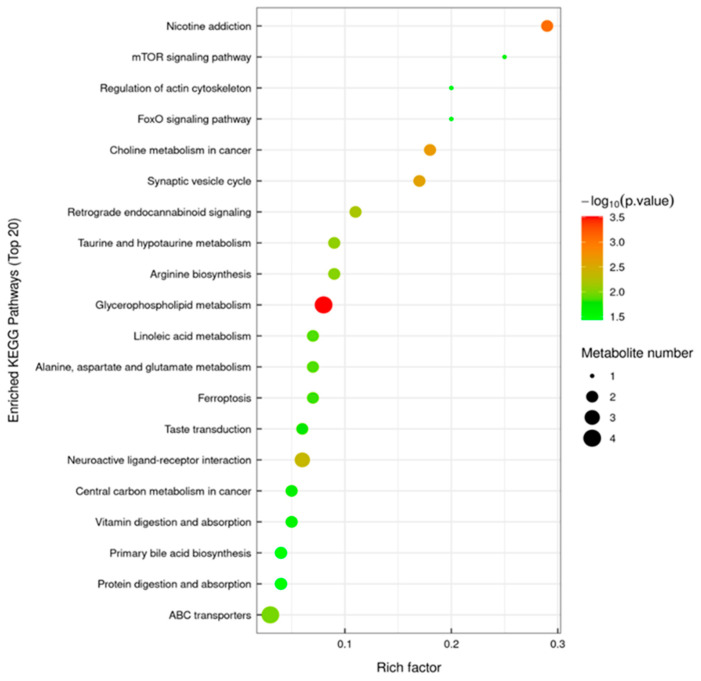
KEGG enrichment pathway of differential metabolites.

The TICs of QC samples showed overlapping peak response intensities and retention times, with well-resolved, sharp peaks, indicating stable instrument performance and minimal technical variation (Figure 7A,B). A total of 1005 metabolites were detected, including 627 in positive ion mode (ESI-) and 378 in negative ion mode (ESI-). Chemical classification analysis revealed that carboxylic acids and their derivatives accounted for the largest proportion (23.48%), followed by glycerophospholipids (12.14%), fatty acyls (7.66%), and unknown metabolites (10.85%) (Figure 7C). PCA results demonstrated good sample distribution and strong model performance (R^2^X > 0.75, PC1 + PC2 + PC3 > 75.3%) (Figure 8). OPLS-DA further highlighted intergroup differences, and seven-fold cross-validation confirmed model reliability (R^2^ > 0.5, Q^2^ > 0.8) (Figure 9). Using VIP > 1 and *p* < 0.05 as selection criteria, 52 significantly altered metabolites were identified, including 35 in positive ion mode (17 upregulated and 18 downregulated) and 17 in negative ion mode (15 upregulated and 2 downregulated) (Table 3, the top 20 differential metabolites are presented; Table 4). Hierarchical clustering analysis revealed clear group-specific clustering patterns for differential metabolites in both ion modes (Figure 10). KEGG pathway enrichment identified 26 significantly perturbed pathways, primarily involving nicotine metabolism, choline metabolism, glycerophospholipid metabolism, and neurotransmitter vesicle cycling (Figure 11), suggesting that these metabolic networks may participate in the cellular response to ZEA.

## 4. Discussion

The goat industry represents a vital component of animal husbandry, and the reproductive capacity of high-quality male breeding stock plays a crucial role in livestock production. Zearalenone, a non-steroidal mycotoxin widely present in animal feed, exhibits reproductive toxicity. Previous studies have demonstrated that ZEA can induce testicular and seminiferous tubule abnormalities, reduce sperm motility, and decrease testosterone secretion [28,29]. LCs are key reproductive cells involved in spermatogenesis and serve as the primary site of steroid hormone synthesis in male animals [30,31]; thus, any toxic effects on LCs may disrupt the spermatogenic process.

Studies have reported that the toxic effects of ZEA are both time- and dose-dependent; apoptosis rates in porcine intestinal epithelial cells (IECs) and goat granulosa cells (GCs) increase significantly with higher ZEA concentrations and prolonged exposure [21,32]. Apoptosis, as a characteristic form of programmed cell death, is critical for male reproductive function. In the present study, ZEA induced dose-dependent death in goat LCs, with a half-maximal inhibitory concentration (IC_50_) of 20 μM, which was subsequently used to explore ZEA-induced male reproductive toxicity. Previous research has shown that ZEA triggers apoptosis via endoplasmic reticulum (ER) stress pathways, upregulating *Caspase-3* and *Caspase-9* expression and increasing the *Bax/Bcl-2* ratio; these cytotoxic effects can be attenuated by the ER stress inhibitor 4-phenylbutyric acid (4-PBA) in embryonic stem cells (ESCs) [19]. Xu et al. [33] reported that ZEA elevates reactive oxygen species (ROS) and cleaved poly ADP-ribose polymerase (PARP) in mouse Sertoli cells, thereby inducing oxidative stress and apoptosis. Consistently, in this study, ZEA treatment increased the expression of *Bcl-2* and *Caspase-3*, and significantly upregulated Bax in goat LCs, indicating that ZEA induces apoptosis in testicular interstitial cells, in agreement with previous findings. Furthermore, decrease in mitochondrial membrane potential is an early hallmark of apoptosis. Xu et al. [34] demonstrated that treatment with 20 μM ZEA for 24 h significantly reduced mitochondrial membrane potential in mouse Sertoli cells, which aligns with the results observed here, suggesting that male reproductive cells across species exhibit conserved responses to ZEA toxicity. Additionally, cell cycle analysis and scratch assay results further confirmed the cytotoxic effects of ZEA on goat LCs.

Testosterone produced by LCs constitutes the primary source of androgens, which, as classical steroid hormones, play essential physiological roles [35]. Zearalenone and its derivatives have been reported to disrupt steroid hormone biosynthesis and alter the expression of key steroidogenic pathway components [36,37]. Steroidogenic acute regulatory protein (*StAR*), the rate-limiting enzyme in steroidogenesis, is inhibited by high ZEA concentrations in LCs, whereas low concentrations may enhance its expression [38]. In this study, ZEA (20 μM) significantly reduced *StAR* expression in goat LCs. *CYP17A1*, *CYP11A1*, and *17β-HSD* are critical genes in the steroidogenic pathway, and changes in their expression can perturb hormone metabolism [39]. ZEA significantly downregulated *CYP17A1*, while *CYP11A1* levels remained unchanged; given its mitochondrial localization, *CYP11A1* stability may be associated with ZEA-induced mitochondrial damage. Notably, *17β-HSD* expression was markedly upregulated. Targeted metabolomics of 18 steroid hormones revealed that ZEA significantly increased 17α-hydroxyprogesterone and corticosterone, markedly elevated progesterone, 21-hydroxyprogesterone, androstenedione, and cortisone, and significantly decreased dihydrotestosterone, with other hormones unchanged. Considering the interconversion of androstenedione and testosterone catalyzed by *17β-HSD*, its upregulation may correlate with elevated androstenedione levels. Collectively, these results indicate that ZEA disrupts both the expression of key steroidogenic genes in goat LCs and steroid hormone synthesis. Given the pathway’s complexity and ZEA’s unique estrogenic activity, further studies are warranted to elucidate the molecular mechanisms underlying these hormonal alterations.

Previous studies have demonstrated that ZEA can induce metabolic disorders by disrupting lactate secretion, lipid synthesis, and other metabolic processes, thereby compromising systemic homeostasis [40,41]. We hypothesize that ZEA similarly affects LCs metabolism. Using untargeted metabolomics and an OPLS-DA model, this study identified 52 significantly altered metabolites, including 6-aminopenicillanic acid, short-chain acylcarnitines, and glycerophospholipids, mainly classified as carboxylic acids and derivatives, glycerophospholipids, and amino acids and derivatives. Pathway enrichment analysis indicated that ZEA primarily perturbs choline metabolism, glycerophospholipid metabolism, and neurotransmitter vesicle cycling in LCs. Glycerophospholipids, as key lipid metabolites, are essential for endocrine homeostasis, and their dysregulation can disrupt cellular physiology [42,43]. We observed that ZEA exposure significantly altered the levels of several glycerophospholipids, including phosphatidylcholine (PC) and phosphatidylethanolamine (PE). Because phospholipids are fundamental components of cellular membranes, their metabolic disturbances can affect the localization and activity of steroidogenic enzymes, thereby impairing the synthesis of steroid hormones such as testosterone [44,45].

This study further revealed significant alterations in multiple amino acid metabolites. Amino acids serve not only as the building blocks for protein synthesis but also participate in physiological processes such as signal transduction, carbohydrate and lipid metabolism, and apoptosis [46]. In particular, glutamate, citrulline, and D-aspartate were upregulated, suggesting that ZEA may disrupt the glutamine–glutamate cycle, potentially inducing neurotoxicity and apoptosis. Long et al. [47] reported that oral ZEA administration in male mice significantly increased alanine aminotransferase (ALT) and aspartate aminotransferase (AST) activities, resulting in suppressed hormone synthesis. KEGG enrichment of neurotransmitter vesicle metabolism supports this mechanism, as glutamate is involved in vesicle release, and its disruption may impair the endocrine function of LCs. Reduced lactate levels indicate that ZEA inhibits glycolysis, leading to decreased ATP production and compromised steroidogenesis. Similarly, in a rat Sertoli cell model, ZEA decreased lactate and pyruvate production and suppressed the expression of glycolytic genes and lactate-producing proteins [48]. KEGG pathway analysis also revealed prominent perturbations in choline metabolism and neurotransmitter vesicle cycling. Choline metabolism is closely linked to phospholipid biosynthesis and acetylcholine production, whereas disruption of neurotransmitter vesicle pathways suggests that ZEA may indirectly impair reproductive function via neuroendocrine mechanisms, consistent with observations in pigs and rats [49,50,51]. Collectively, these findings indicate that ZEA exerts toxic effects on goat LCs by disrupting metabolic pathways and impairing steroid hormone synthesis, corroborating the observed structural damage and hormonal alterations.

## 5. Conclusions

This study employed goat LCs as the experimental model. The results demonstrated that ZEA induces morphological alterations in LCs and damages subcellular structures, including mitochondria and the endoplasmic reticulum. ZEA also decreases mitochondrial membrane potential, arrests the cell cycle, and exerts cytotoxic effects by inducing oxidative stress, disrupting metabolic homeostasis, and impairing steroid hormone synthesis. Collectively, the above results all confirm that ZEA has multiple harmful effects on the reproductive cells of male goats.

However, this study only investigated the toxic effects of ZEA at the cellular level and did not conduct research at the animal level. In the future, research on ZEA in animals can be carried out, in addition, based on this study, economically effective natural antagonists of ZEA can be discovered to counteract its toxic effects.

## Figures and Tables

**Table 1:** Primer information.

Gene	Prime Sequence	Size (bp)	Tm (°C)
*Bcl*-2	F: ATGTGTGTGGAGAGCGTCA R: AGAGACAGCCAGGAGAAATC	182	60
*Bax*	F: CATCGGAGATGAATTGGACAGTAA R: GGCCTTGAGCACCAGTTTGC	178	60
*Caspase 3*	F: CATTATTCAGGCCTGCCGAG R: CTCGAGCTTGTGAGCGTACT	220	58
*StAR*	F: GCGACCAAGAGCTTGCCTATATCC R: CTCTCCTTCTTCCAGCCCTCCTG	94	57
*CYP17A1*	F: GGCCCAAGACCAAGCACTC R: GGAACCCAAACGAAAGGAATAG	161	60
*CYP11A1*	F: ATGGCTCCAGAGGCAATAAA R: AAGGCAAAGTGAAACAGGTC	146	56
*17β-HSD*	F: GGCGGTCCTCGTGGTCCTATAC R: TGGTTCCCGAAGCCTGAGTCAC	117	58
*β-actin*	F: CTGAGCGCAAGTACTCCGTGT R: GCATTTGCGGTGGACGAT	125	60

Note: F stands for upstream primer, and R stands for downstream primer.

**Table 2:** Effect of ZEA on steroid hormone content.

Name	Standard Curve Equation	CK	ZEA	SEM	*p*
17α-Hydroxyprogesterone (pg/mL)	y = 0.9x − 0.000396	2.53 ^b^	5.10 ^a^	0.0007	<0.05
Corticosterone (pg/mL)	y = 0.452x − 0.00676	19.07 ^b^	24.10 ^a^	0.0018	<0.05
5-α-Dihydrotestosterone (pg/mL)	y = 0.246x − 0.00172	16.40 ^a^	13.27 ^b^	0.0005	<0.05
Pregnenolone (pg/mL)	y = 0.105x − 0.000895	10.87	11.37	0.0029	>0.05
Progesterone (pg/mL)	y = 0.384x − 0.000954	4.00 ^b^	5.50 ^a^	0.0003	<0.05
11-Deoxycortisol (pg/mL)	y = 0.77x − 0.000355	1.57	2.27	0.0003	>0.05
11-Deoxycorticosterone (pg/mL)	y = 0.995x + 0.00039	0.06 ^b^	0.57 ^a^	0.0001	<0.05
Androstenedione (pg/mL)	y = 1.06x + 0.0006	16.70 ^b^	27.20 ^a^	0.0014	<0.05
Cortisone (pg/mL)	y = 0.649x − 0.000319	2.81 ^b^	4.40 ^a^	0.0001	<0.05
Testosterone (pg/mL)	y = 0.584x + 0.000157	2.11	2.00	0.0002	>0.05
Cortisol (pg/mL)	y = 0.465x + 0.000628	7.83	20.10	0.0091	>0.05
Dehydroepisndrosterone (pg/mL)	y = 0.243x + 0.00264	34.85	40.37	0.0065	>0.05
Estradiol (pg/mL)	y = 0.852x − 0.00285	5.05	3.73	0.0006	>0.05
Aldosterone (pg/mL)	y = 0.271x + 0.00109	0.0002	0.0036	0.0013	>0.05
Estriol (pg/mL)	y = 0.628x + 0.000594	2.46	2.73	0.0021	>0.05
Estrone (pg/mL)	y = 0.663x + 5.21	0.31	0.37	0.00004	>0.05
17A-Hydroxypregnenolone (pg/mL)	y = 0.256x + 0.00146	3.43	1.57	0.0001	>0.05
Dehydroepiandrosterone Sulfate (ng/mL)	y = 0.011x − 0.0116	1.16	1.17	0.0065	>0.05

Note: Different lowercase letters within a row indicate significant differences among steroid hormone content in goat LCs. (a,b)—significant differences (*p* < 0.05) relative to the untreated cells.

**Table 3:** Significant metabolites on positive ion mode.

Number	Name	Fold Change	*p*	VIP
1	(+)-6-aminopenicillanic acid	0.84	0.04	4.42
2	(r)-butyrylcarnitine	0.29	0.00	3.25
3	1-(1z-hexadecenyl)-sn-glycero-3- 2-phosphocholine	0.41	0.03	1.24
4	1-(1z-octadecenyl)-2-(9z-octadecenoyl)- 2-sn-glycero-3-phosphocholine (18:0/18:1)	1.26	0.01	4.16
5	1-hexadecanoyl-2-octadecadienoyl-sn- 2-glycero-3-phosphocholine (16:0/16:2)	1.22	0.00	5.31
6	1-hexadecyl-2-(8z,11z,14z-eicosatrienoyl)-sn-glycero-3-phosphocholine (16:0/20:3)	1.24	0.00	2.93
7	1-hexadecyl-2-(9z-octadecenoyl)-sn- 2-glycero-3-phosphocholine (16:0/18:1)	1.18	0.01	7.22
8	1-hexadecyl-sn-glycero-3-phosphocholine (O-16:0/0:0)	0.36	0.05	3.22
9	1-oleoyl-2-palmitoyl-sn-glycero-3- 2-phosphocholine (18:1/16:0)	0.92	0.04	4.71
10	1-palmitoyl-2-lauroyl-sn-glycero-3- phosphorylcholine	1.34	0.00	1.99
11	1,2-dihexadecanoyl-sn-glycero-3- Phosphocholine (16:0/16:0)	0.80	0.00	5.33
12	1,2-dipalmitoleoyl-sn-glycero-3- Phosphocholine (16:1/16:1)	1.41	0.00	2.57
13	18-β-glycyrrhetic acid methyl ester	0.17	0.00	2.25
14	4-hydroxy-1-(2-hydroxyethyl)-2,2,6,6- tetramethylpiperidine	0.21	0.00	13.03
15	7-α-hydroxy-4-cholesten-3-one	2.74	0.00	2.60
16	Acetylcholine	0.54	0.00	1.43
17	Citrulline	2.00	0.05	1.04
18	Diethanolamine	0.42	0.01	1.75
19	Dimethyl phthalate	0.60	0.02	1.21
20	Glutamic acid	1.16	0.00	1.16

**Table 4:** Significant metabolites on negative ion mode.

Number	Name	Fold Change	*p*	VIP
1	1-palmitoyl-2-linoleoyl-sn-glycero-3-phospho-(1′-rac-glycerol) (16:0/18:2)	1.85	0.00	1.51
2	1-stearoyl-2-linoleoyl-sn-glycero-3-phosphoethanolamine (18:0/18:2)	1.27	0.01	1.57
3	2,4-di-tert-butylphenol	1.23	0.04	2.43
4	6-Hydroxydopamine	1.45	0.00	1.07
5	Cis-7,10,13,16-docosatetraenoic acid	0.63	0.02	1.91
6	Cis-9-palmitoleic acid	1.48	0.01	2.85
7	D-aspartic acid	2.25	0.00	1.06
8	Lactate acid	0.44	0.00	1.97
9	Glutamic acid	1.30	0.01	1.44
10	Linoleic acid	1.15	0.01	1.32
11	N-benzyloxycarbonylglycine	1.32	0.03	2.79
12	Pantothenate	1.78	0.00	3.97
13	PC (18:1/9)	1.14	0.03	1.37
14	PG (36:2)	2.03	0.00	4.82
15	PG (36:3)	1.62	0.01	1.98
16	Taurine	1.91	0.01	1.48
17	Zearalenone	55.25	0.00	4.80

Note: “Name” represents the name of the metabolite; “VIP” represents the variable projection importance. The larger the value, the more important the variable is.; “Fold change” indicates the difference ratio. A fold change greater than 1 indicates an increase, while a fold change less than 1 indicates a decrease.; “*p*” represents significance analysis. The smaller the “*p*” value, the more significant the difference.

## Data Availability

The original contributions presented in the study are included in the articles as Supplementary Material. Further inquiries can be directed to the corresponding author.

## Supplementary Materials

The following supporting information can be downloaded at: https://www.mdpi.com/article/10.3390/ani16020283/s1.
